# Relationship Between Mast Cell Population of Microenvironment and Prognosis in Colorectal Cancer

**DOI:** 10.3390/jcm14238312

**Published:** 2025-11-22

**Authors:** Neşe Yeldir, Ebru Engin Delipoyraz, Aslı Çakır, Ahmet Bilici

**Affiliations:** 1 Department of Pathology, Istanbul Medipol University, Istanbul 34200, Turkey; acakir@medipol.edu.tr; 2 Department of Medical Oncology, Istanbul Medipol University, Istanbul 34200, Turkey; drebruengin@gmail.com (E.E.D.); ahmet.bilici@medipol.com.tr (A.B.)

**Keywords:** colorectal cancer, mast cell tryptase, tumor microenvironment, prognosis, immunohistochemistry

## Abstract

**Background:** Mast cells are integral components of the tumor microenvironment and have been implicated in the regulation of tumor progression in various malignancies. The association between inflammation and colorectal cancer (CRC) development has become increasingly recognized. Depending on the tumor microenvironment, mast cells may exert either pro-tumorigenic or antitumorigenic functions. **Objective:** This study aimed to evaluate the relationship between stromal mast cell density and prognostic factors in patients with CRC. **Methods:** In this retrospective cohort study, 81 patients who underwent curative surgical resection for CRC were analyzed. Immunohistochemical staining for mast cell tryptase (MCT) was performed on paraffin-embedded tumor specimens. Mast cells were quantified in regions of hot spots within the tumor stroma. Patients were categorized as high mast cell density (MCC-H, ≥22 cells/HPF) or low mast cell density (MCC-L, <22 cells/HPF). Associations with clinicopathological parameters were assessed using chi-square or Fisher’s exact tests. Progression-free survival (PFS) and overall survival (OS) were analyzed using Kaplan–Meier estimates and log-rank tests. Independent prognostic factors were identified using multivariate Cox proportional hazards regression, with hazard ratios (HRs) and 95% confidence intervals (CIs) reported. **Results:** ROC analysis identified an MCC cut-off of 22 cells/HPF (AUC = 0.61; sensitivity = 0.67, specificity = 0.52) for mortality prediction. Multivariate analysis revealed lymph node involvement (HR: 1.41, 95% CI: 1.03–1.94, *p* = 0.033) and macroscopic tumor perforation (HR: 0.15, 95% CI: 0.04–0.55, *p* = 0.004) as independent predictors of PFS. High MCC (≥22) independently predicted improved OS (HR: 0.07, 95% CI: 0.006–0.87, *p* = 0.039). A significant association was observed between OS, MCC, and lymph node stage. **Conclusions**: Stromal mast cell count is an independent prognostic factor for overall survival in patients with CRC. Our findings suggest that MCC may serve as a reliable prognostic biomarker following surgical resection and could aid in postoperative risk stratification.

## 1. Introduction

Mast cells are innate immune effector cells derived from bone marrow progenitors that migrate to and reside within peripheral tissues. They play a critical role in orchestrating inflammatory responses through the release of bioactive mediators, including cytokines and chemokines, particularly during allergic and hypersensitivity reactions [1,2]. Beyond their classical role in allergic inflammation, increased mast cell density has been documented in several malignancies and is implicated in both innate and adaptive immune regulation [3].

In the gastrointestinal tract, mast cells participate in diverse biological processes, ranging from host defense against enteric infections to modulation of allergic responses and tumorigenesis [4]. Colorectal cancer (CRC) is among the most prevalent malignancies worlwide and remains a leading cause of cancer-related mortality. While pathological stage is considered the most reliable prognostic indicator in CRC [5]; additional histopathological features—including histological grade, lymphovascular invasion, macroscopic tumor perforation, perineural invasion, lymph node metastasis, tumor budding, and tumor nodules—also hold significant prognostic value [6].

Recently, the role of the tumor microenvironment (TME) in cancer progression and prognosis has gained increasing recognition. Within the TME, mast cells are key regulators that influence tumor growth and behavior through their involvement in processes such as cell proliferation, angiogenesis, invasion, and metastasis [7,8]. However, studies evaluating the prognostic significance of mast cells in CRC have reported conflicting results: some indicate that high mast cell density is associated with poor prognosis, while others suggest a protective effect [9]. These discrepancies likely reflect methodological heterogeneity, including differences in counting methods, cut-off values, and cohort characteristics.

Given these gaps, there is a clear need for well-defined, CRC-specific studies that apply standardized methodologies to clarify the prognostic role of mast cells. The present study aims to systematically evaluate tryptase-positive mast cells in a defined CRC cohort, applying standardized counting protocols, and to correlate stromal mast cell density with clinicopathological parameters as well as progression-free and overall survival outcomes. Through this approach, we aim to provide a more robust assessment of the potential independent prognostic value of mast cells in CRC.

## 2. Methods

### 2.1. Patients and Tissue Samples

#### 2.1.1. Patients and Tissue Samples

This retrospective study included patients diagnosed with CRC between May 2016 and June 2024 at the Department of Pathology, Istanbul Medipol University. Demographic information (age and sex), along with clinical and histopathological data including tumor location, size, grade, stage, macroscopic perforation, lymphovascular invasion, and perineural invasion—were retrieved from pathology reports and the institutional electronic medical record system. Patients with complete clinical and pathological data were included, whereas those lacking adequate records or available paraffin-embedded tissue blocks were excluded. Representative formalin-fixed, paraffin-embedded (FFPE) tissue blocks containing both tumor and adjacent normal mucosa were obtained from the pathology archives and re-evaluated. Pathological parameters assessed included tumor location, pathological stage, histological grade, maximum tumor diameter, macroscopic perforation, lymphovascular invasion, perineural invasion, lymph node metastasis, tumor budding, tumor nodules, probability of microsatellite instability, and mucinous morphology. Clinical parameters, such as the presence of distant metastasis, clinical stage, treatment modalities, therapeutic response, and follow-up outcomes (progression and survival), were also analyzed. Progression-free survival (PFS) was defined as the time interval from diagnosis to the first occurrence of relapse, metastasis, death, or last follow-up. Overall survival (OS) was defined as the time from diagnosis to death from any cause.

#### 2.1.2. Immunohistochemical Method

Immunohistochemical staining for mast cell tryptase (MCT) was performed on selected FFPE tissue sections. Four-micron-thick sections were prepared from formalin-fixed tumor blocks and subjected to MCT staining (Rabbit Monoclonal Clone G3, Cell Marque Corporation, Rocklin, CA, USA) using the Ventana Benchmark Ultra automated immunostaining platform. A 1:500 dilution of the primary antibody was used. Appropriate positive and negative controls were included in each staining run to ensure accuracy and reproducibility, with colon tissue serving as the positive control.

#### 2.1.3. Histopathological Evaluation

Hematoxylin and eosin (H&E)-stained slides were reviewed by an experienced pathologist to confirm diagnosis and identify representative tumor areas. MCT-stained slides were then examined, and mast cells exhibiting dark, granular cytoplasmic staining were considered positive. The number of MCT-positive mast cells was counted in the areas showing the highest mast cell density within the tumor stroma. To minimize potential bias in selecting areas of highest mast cell density, entire tissue sections were scanned at low magnification, and multiple regions were evaluated before selecting the hot-spot areas for detailed counting. Mast cell counts were performed on tissue sections by two independent pathologists simultaneously to assess interobserver variability. In addition, intraobserver variability was evaluated by having one of the pathologists repeat the counts at two separate time points. Counts were conducted carefully to ensure consistency and reliability of the observations. The resulting mast cell count (MCC) was recorded for each case and used for subsequent statistical analysis.

The raw counts of mast cells per case are provided in Supplementary Table S1.

### 2.2. Statistical Analysis

All statistical analyses were conducted using IBM SPSS Statistics, version 27.0 (IBM Corp., Armonk, NY, USA). Continuous variables were expressed as median (range) or mean ± standard deviation (SD), and categorical variables were presented as absolute numbers and percentages.

**Figure 1:**
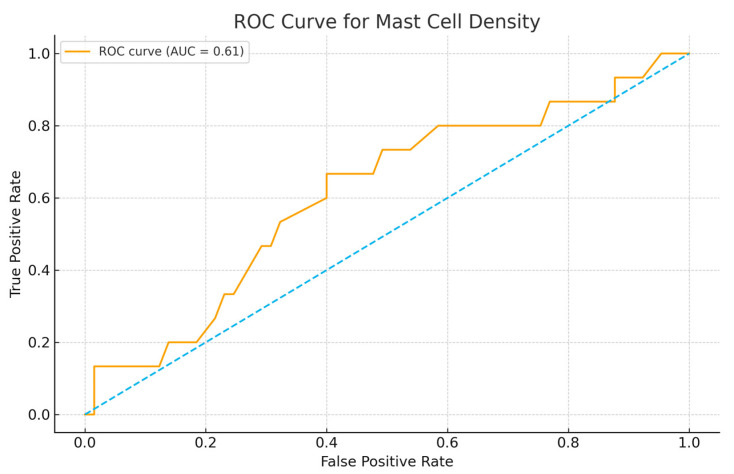
ROC curves for MCT predicting mortality.

Receiver operating characteristic (ROC) analysis was performed to evaluate the discriminatory performance of mast cell count (MCC) value in predicting mortality. MCC demonstrated a modest predictive ability with an area under the ROC curve (AUC) of 0.61. Using a predefined cut-off value of 22, the sensitivity was 0.67 and the specificity was 0.52 for identifying patients who experienced the event. The ROC curve and the corresponding threshold are presented in Figure 1. Although the biomarker alone shows limited discriminatory power, the results suggest a potential association between higher mast cell density and outcomes, warranting further validation in larger cohorts or in combination with additional prognostic variables. Cases with an MCC ≥ 22 were categorized as high mast cell density (MCC-H), whereas those with an MCC < 22 were categorized as low mast cell density (MCC-L). Associations between MCT density and clinicopathological or prognostic parameters were assessed using the chi-square test or Fisher’s exact test, as appropriate. PFS and OS were estimated using the Kaplan–Meier method, and intergroup differences were evaluated with the log-rank test.

To determine independent prognostic factors influencing PFS and OS, variables significant in univariate analysis were subsequently included in a multivariate Cox proportional hazards regression model. Results were expressed as hazard ratios (HR) with corresponding 95% confidence intervals (CI). The proportional hazards (PH) assumption for all Cox regression models was evaluated using Schoenfeld residuals, including both global tests and covariate-specific assessments. Visual inspection of log–log survival plots was also performed to confirm proportionality. No significant violations of the PH assumption were detected (global Schoenfeld test *p* > 0.05), indicating that the Cox models were appropriate for the analysis. A two-tailed *p*-value of <0.05 was considered statistically significant.

## 3. Results

A total of 81 patients diagnosed with CRC were included in the study. The cohort comprised 55 males (67.9%) and 26 females (32.1%), with a median age of 60 years (range, 26–77 years). At diagnosis, 60.5% of patients presented with early-stage disease, while 39.5% had metastatic disease. Microsatellite instability (MSI) status was high in 3.7% of cases and low in 96.3%. Histopathological examination revealed mucinous morphology in 19.8% of tumors, and macroscopic tumor perforation was identified in 3.7% of patients.

The most frequent metastatic site at presentation was the liver (28.4%), followed by the peritoneum (7.4%), lung (4.9%), and brain (1.2%). Among the 38 patients with available RAS/RAF molecular data, KRAS mutations were detected in 57.9%, NRAS mutations in 5.3%, BRAF mutations in 2.6%, while 34.2% were wild-type for all three genes.

**Figure 2:**
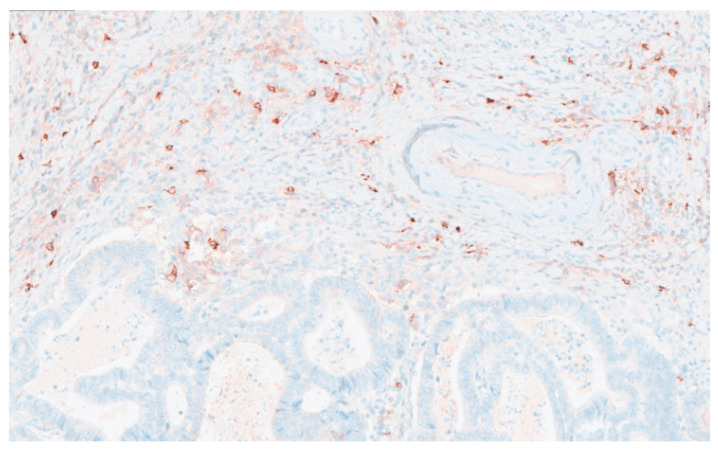
Area with high stromal mast cell density in colorectal tumor tissue. Mast cells were visualized by tryptase immunostaining. Scale bar = 50 µm. Original magnification ×40.

**Figure 3:**
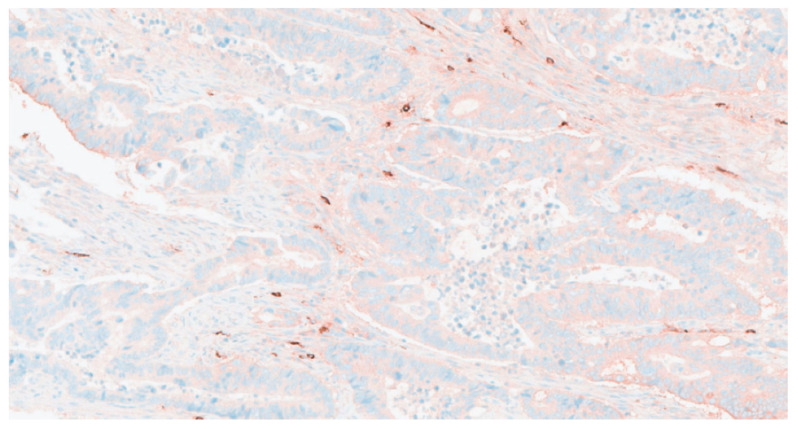
Area with low stromal mast cell density in colorectal tumor tissue. Mast cells were visualized by tryptase immunostaining. Scale bar = 50 µm. Original magnification ×40.

When patients were stratified according to MCC into high-density (MCC-H, *n* = 41) (Figure 2) and low-density (MCC-L, *n* = 40) (Figure 3) groups, no statistically significant associations were observed between MCC category and demographic or clinicopathological variables, including age, sex, tumor stage, anatomical localization, histological grade, pT classification, vascular invasion, perineural invasion, lymphatic invasion, or tumor nodules. Notably, a significant correlation was identified between MCC category and lymph node grouping (*p* = 0.039) (Table 1).

In the univariate analysis for PFS, several variables were found to be significantly associated with prognosis, including metastatic disease at diagnosis, presence of liver metastases, lymph node stage, MCC, macroscopic tumor perforation, and perineural invasion (*p* < 0.05 for all).

However, in the multivariate Cox proportional hazards model, only lymph node stage (HR = 1.41; 95% CI, 1.03–1.94; *p* = 0.033) and macroscopic tumor perforation (HR = 0.15; 95% CI, 0.04–0.55; *p* = 0.004) emerged as independent prognostic factors for PFS.

**Figure 4:**
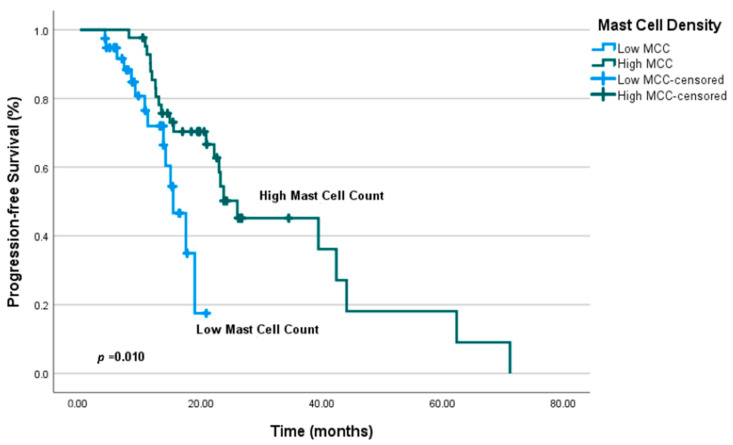
Progression-free survival (PFS) curves according to mast cell count (MCC) categories. Kaplan–Meier analysis was used to compare survival between high and low MCC groups.

While the MCC category demonstrated a statistically significant association with PFS in the univariate analysis (*p* = 0.01) (Figure 4), this significance was not retained in the multivariate analysis (*p* ≥ 0.05). Detailed results of the univariate and multivariate analyses for PFS are presented in Table 2.

**Figure 5:**
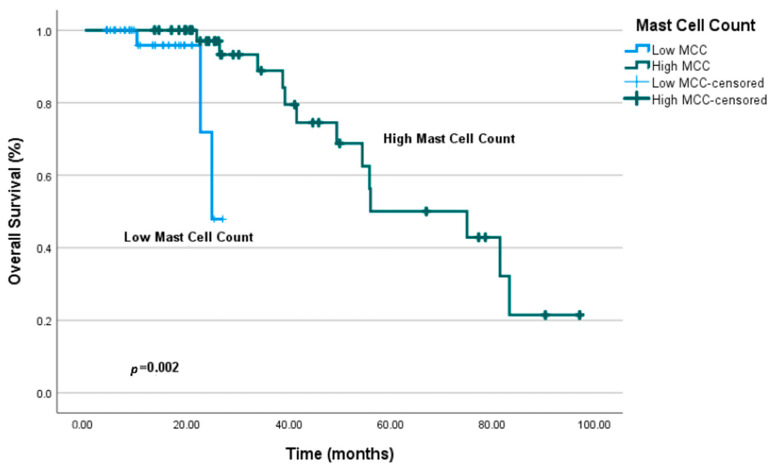
Overall survival (OS) curves according to mast cell count (MCC) categories. Kaplan–Meier analysis demonstrates differences in OS between high and low MCC groups.

In univariate analysis of OS, significant associations were identified between MCC category and lymph node stage. Patients with high MCC (≥22) had a median OS of 74.9 months (95% CI: 41.1–108.8), whereas patients with low MCC (<22) had a median OS of 24.8 months, a difference that was statistically significant (*p* = 0.002) (Figure 5). Lymph node stage was also significantly associated with OS in univariate analysis (*p* = 0.006).

In multivariate Cox regression analysis, only the MCC category emerged as an independent prognostic factor for OS (HR = 0.07; 95% CI, 0.006–0.87; *p* = 0.039). Other clinicopathological variables—including stage at diagnosis, liver metastasis, macroscopic tumor perforation, tumor location, histological grade, pT classification, perineural, lymphatic, or venous invasion, tumor budding, and tumor nodules—were not significantly associated with OS (Table 3).

## 4. Discussion

The TME is primarily composed of non-neoplastic inflammatory cells, including lymphocytes, macrophages, and mast cells, a characteristic feature of many solid tumors, including CRC [10]. Despite their prevalence, the prognostic significance of these inflammatory cells in CRC remains unclear. In this study, we specifically investigated the impact of intratumoral MCC on survival outcomes in CRC patients.

Mast cells are key cellular component of the TME, and their density and functional activity have been evaluated across various malignancies. Emerging evidence highlights a link between inflammation and CRC development, underscoring the potential role of mast cells in tumorigenesis [11]. Mast cells have been implicated in tumor-associated angiogenesis, and their secreted mediators may contribute to metastatic progression in CRC [12].

In our cohort, increased MCC was significantly associated with improved survival outcomes. Among other prognostic factors, lymph node involvement showed a significant relationship with MCC, with mast cell density differing according to nodal stage. This finding is consistent with observations from studies using larger and more homogeneous patient cohorts, suggesting that MCC may reflect the immune landscape of the tumor [9].

The prognostic implications of mast cells are complex and appear to vary across cancer types. In certain malignancies, such as head and neck squamous cell carcinoma, increased mast cell density has been linked to poor outcomes [13,14,15,16], whereas in other tumors-including esophegeal, renal, transitional cell, breast, cervical, and prostate cancers- high mast cell density has been associated with favorable outcomes [17,18,19,20,21,22,23]. These discrepancies highlight the context-dependent role of mast cells, influenced by tumor type, spatial localization within the TME, and methodological differences in mast cell assessment.

Although historically less emphasized, mast cells can exert antitumor functions. They modulate adaptive immune responses, enhance antitumor T cell activity, can shape angiogenesis and stromal remodeling in ways that inhibit tumor progression. For instance, analyses of The Cancer Genome Atlas revealed that mast cell infiltration in adrenocortical carcinoma was predominantly associated with antitumor activity [24].

In the present study, MCC-H emerged as a favorable prognostic factor for OS and PFS, supporting the notion that mast cells can exert antitumor effects. Although mast cells have been implicated in promoting tumor angiogenesis and facilitating invasion and metastasis [25], they also play important roles in shaping adaptive immune responses and can enhance antitumor T cell activity [26]. These dual functions suggest that the prognostic significance of mast cells depends on multiple factors, including tumor type, the methodological approach used for their assessment, and their spatial distribution within the TME.

In the multivariate analysis, MCC-H was associated with improved OS but not with PFS. This divergence is biologically plausible, as mast cells exert not only rapid activation but also a gradual form of activation characterized by the slow release of specific mediators leading to chronic inflammatory and local tissue changes [27]. Such delayed immunomodulatory effects are unlikely to influence early radiologic progression, but they may progressively reshape the tumor microenvironment over time, thereby exerting a stronger impact on long-term overall survival.

This study has several limitations that should be acknowledged. First, its retrospective design, modest sample size, and single institution setting may limit generalizability and introduce potential selection bias. Therefore, the proposed cut off value should be interpreted with caution and may not be directly generalizable to other populations. Second, we did not directly assess T-cell infiltration or other immune cell populations, raising the possibility that the observed association between stromal mast cell density and survival partly reflects broader antitumor immune activity rather than mast cell–specific effects. Third, only tryptase-positive mast cells were evaluated, which may not capture the full spectrum of mast cell heterogeneity.

Future studies using larger, multicenter cohorts and comprehensive immune profiling—including interactions between mast cells and other immune cells—are warranted to validate our findings and clarify the mechanistic basis of mast cell–mediated effects in CRC. Collectively, our results suggest that MCC may serve as a valuable prognostic biomarker, potentially aiding postoperative risk stratification in colorectal cancer patients [28].

## 5. Conclusions

In this study, MCC-H was identified as a favorable prognostic factor for both OS and PFS in CRC patients. It is possible that results may differ in studies involving larger and more diverse patient cohorts. Although the prognostic significance of MCC remains a matter of debate, our findings indicate that MCC may serve as an independent prognostic biomarker and could provide a foundation for future investigations into mast cell related therapeutic strategies.

## Figures and Tables

**Table 1:** The relationship of mast cell count with clinicopathological features.

Factor	MCC-High (*n* = 41)	MCC-Low (*n* = 40)	*p* Value
**Age, years, *n* (%)**			0.82
<60	19 (46.3)	20 (50)	
≥60	22 (53.7)	20 (50)	
**Sex, *n* (%)**			0.05
Female	9 (22)	17 (42.5)	
Male	32 (78)	23 (57.5)	
**Stage at diagnosis, *n* (%)**			0.25
Early	22 (53.7)	27 (67.5)	
Metastatic	19 (46.3)	13 (32.5)	
**Localization**			0.80
Left colon	29 (70.7)	30 (75)	
Right colon	12 (29.3)	10 (25)	
**Grade, *n* (%)**			0.10
1	19 (46.3)	11 (27.5)	
2	18(43.9)	27 (67.5)	
3	4 (9.8)	2 (5%)	
**pT group, *n* (%)**			0.54
T2	2 (4.9)	4 (10)	
T3	20 (48.8)	23 (57.5)	
T4a	13 (31.7)	8 (20)	
T4b	6 (14.6)	5 (12.5)	
**Lymp node group, *n* (%)**			0.039 *
N0	18 (43.9)	16 (40)	
N1a	10 (24.4)	2 (5)	
N1b	9 (22)	13 (32.5)	
N1c	1 (2.4)	2 (5)	
N2a	3 (7.3)	2 (5)	
N2b	0	5 (12.5)	
**Stage, ** ***n* (%)**			0.49
**I**	1 (2.4)	1 (2.5)	
**II**	9 (22)	8 (20)	
**III**	12 (29.3)	18 (45)	
**IV**	19 (46.3)	13 (32.5)	
**Lymphatic invasion, *n* (%)**			1
Yes	16 (39)	15 (37.5)	
No	25 (61)	25 (62.5)	
**Venous invasion, *n* (%)**			0.82
Yes	16 (39)	17 (42.5)	
No	25 (61)	23 (57.5)	
**Perineural invasion, *n* (%)**			0.49
Yes	13 (31.7)	16 (40)	
No	28 (68.3)	24 (60)	
**Tumor budding, *n* (%)**			0.66
No	17 (41.5)	12 (30)	
Low	14 (34.1)	17 (42.5)	
Medium	7 (17.1)	9 (22.5)	
High	3 (7.3)	2 (5)	
**Tumor nodule, *n* (%)**			0.54
Yes	14 (34.1)	12 (30)	
No	26 (63.4)	28 (70)	

* indicates statistically significant values (*p* < 0.05).

**Table 2:** The results of univariate and multivariate analysis for PFS.

Factors	Median PFS (Months)	Univariate *p* Value	HR (95% CI)	Multivariate *p* Value
**Stage at diagnosis**		0.001 *	2.63 (0.96–7.22)	0.061
Early	39.4			
Metastatic	14.8			
**Initial liver metastasis**		0.017 *	0.57 (0.19–1.72)	0.317
Yes	13.4 (10–16.8)			
No	26 (8.8–43.2)			
**MCC group**		0.01 *	1.18 (0.41–3.44)	0.760
≥22	26 (12.2–39.8)			
<22	15.4 (12.3–18.4)			
**Macroscopic tumor perforation**		0.005 *	0.15 (0.04–0.55)	0.004 *
Yes	11.8 (2.5–21.1)			
No	23.2 (20–26.3)			
**Perineural invasion**		0.003 *	0.50 (0.22–1.17)	0.111
Yes	15.4 (13.3–17.4)			
No	26 (5.8–46.2)			
**Lymphatic invasion**		0.23		
Yes	15.4 (12–18.7)			
No	26 (19.7–32.3)			
**Venous invasion**		0.22		
Yes	15.4 (12.1–18.6)			
No	23.2 (18.6–27.8)			
**Tumor budding**		0.32		
None	26 (9.2–42.8)			
Low	18.9 (9–28.9)			
Median	23.2 (14.8–31.6)			
High	11.6 (9.7–13.4)			
**Tumor nodule**		0.023 *	0.48 (0.21–1.48)	0.88
Yes	14.1 (11.4–16.8)			
No	23.2 (9.9–36.5)			
**Localization**		0.38		
Right colon	39.4 (0–90.6)			
Left colon	22.1 (18–26.3)			
**Grade**		0.38		
1	22.1 (8.7–35.5)			
2	23.2			
3	11.6 (0–27.4)			
**pT group**		0.17		
T2	26 (18.5–33.5)			
T3				
T4a	15.4 (12.3–18.5)			
T4b	13 (0.9–25.1)			
**Lymph node group**		0.026 *	1.41 (1.03–1.94)	0.033 *
N0	39.4 (9.5–69.3)			
N1a	14.8 (11.8–17.8)			
N1b	17.5 (10.6–24.3)			
N1c				
N2a	10.7			
N2b	13.7 (2.3–25.1)			

PFS: Progression free survival, MCC: Mast cell count, HR: Hazard ratios, CI: Confidence intervals. * indicates statistically significant values (*p* < 0.05).

**Table 3:** Univariate and multivariate analysis for OS.

Factors	Median OS (Months)	Univariate *p* Value	HR (95% CI)	Multivariate *p* Value
**Stage at diagnosis**		0.29	2.15 (0.47–9.74)	0.32
Early	74.9 (26.3–123.5)			
Metastatic	49.4 (33–65.7)			
**Initial liver metastasis**		0.64	1.10 (0.26–4.55)	0.89
Yes				
No	56 (25.9–86.1)			
**MCC group**		0.002 *	0.07 (0.006–0.87)	0.039 *
≥22	74.9 (41.1–108.8)			
<22	24.8			
**Macroscopic tumor perforation**		0.66		
Yes	81.4			
No	55.8 (52.8–58.7)			
**Perineural invasion**		0.15		
Yes	55.8 (40.7–70.9)			
No	74.9 (43.9–105.9)			
**Lymphatic invasion**		0.91		
Yes	55.8 (30.6–81)			
No	56 (36.7–75.3)			
**Venous invasion**		0.53		
Yes	55.8 (31.4–80.1)			
No	56 (29.2–82.8)			
**Tumor budding**		0.13		
None	74.9 (48.6–101.2)			
Low	56 (32.1–79.8)			
Median	33.8			
High	38.8			
**Tumor nodule**		0.30		
Yes	49.4 (18.5–80.2)			
No	56 (32.9–79)			
**Localization**		0.91		
Right colon	54.4 (41.9–66.8)			
Left colon	81.4 (54.1–108.7)			
**Grade**		0.08		
1	74.9 (42.1–107.8)			
2				
3	55.8 (27.9–83.7)			
**pT group**		0.88		
T2	83.3			
T3				
T4a	56 (32–80)			
T4b	55.8 (33.4–78.2)			
**Lymph node group**		0.006 *	1.45 (0.86–2.44)	0.16
N0				
N1a				
N1b				
N1c				
N2a				
N2b				

OS: Overall survival, MCC: Mast cell count, HR: Hazard ratios, CI: Confidence intervals. * indicates statistically significant values (*p* < 0.05).

## Data Availability

No new data were created or analyzed in this study. Therefore, data availability is not applicable.

## Supplementary Materials

The following supporting information can be downloaded at: https://www.mdpi.com/article/10.3390/jcm14238312/s1, Table S1: Raw counts of mast cells per case.
