# Supplementary material for: Relationship Between Mast Cell Population of Microenvironment and Prognosis in Colorectal Cancer
Source: J Clin Med. 2025 Nov 22;14(23):8312. doi: 10.3390/jcm14238312 (PMC12693393; doi:10.3390/jcm14238312)
Supplement: Supplementary file 1 [file jcm-14-08312-s001.zip › jcm-3978203-supplementary.pdf]

Supplementary Table S1 – Raw counts of mast cells per case

Case number	Mast cell counts in the area of the tumor stroma with the highest mast cell density	Case number	Mast cell counts in the area of the tumor stroma with the highest mast cell density
**1**	12	**42**	30
**2**	20	**43**	45
**3**	7	**44**	30
**4**	13	**45**	56
**5**	18	**46**	42
**6**	12	**47**	58
**7**	16	**48**	57
**8**	6	**49**	35
**9**	16	**50**	35
**10**	15	**51**	38
**11**	9	**52**	52
**12**	8	**53**	108
**13**	18	**54**	22
**14**	3	**55**	55
**15**	30	**56**	50
**16**	12	**57**	33
**17**	15	**58**	45
**18**	22	**59**	53
**19**	17	**60**	30
**20**	14	**61**	48
**21**	18	**62**	70
**22**	16	**63**	40
**23**	10	**64**	21
**24**	16	**65**	20
**25**	16	**66**	30
**26**	7	**67**	40
**27**	20	**68**	7
**28**	8	**69**	35
**29**	18	**70**	28
**30**	11	**71**	35
**31**	32	**72**	17
**32**	25	**73**	68
**33**	16	**74**	35
**34**	11	**75**	13
**35**	6	**76**	32
**36**	24	**77**	38
**37**	11	**78**	40
**38**	30	**79**	48
**39**	21	**80**	35
**40**	25	**81**	8
**41**	36		
